# High-speed Intravascular Photoacoustic Imaging of Lipid-laden Atherosclerotic Plaque Enabled by a 2-kHz Barium Nitrite Raman Laser

**DOI:** 10.1038/srep06889

**Published:** 2014-11-04

**Authors:** Pu Wang, Teng Ma, Mikhail N. Slipchenko, Shanshan Liang, Jie Hui, K. Kirk Shung, Sukesh Roy, Michael Sturek, Qifa Zhou, Zhongping Chen, Ji-Xin Cheng

**Affiliations:** 1 Weldon School of Biomedical Engineering, Purdue University, West Lafayette, 47906, USA; 2 Department of Biomedical Engineering, NIH Ultrasonic Transducer Resource Center, University of Southern California, Los Angeles, California 90089, USA; 3 Spectral Energy, LLC, Dayton, Ohio, 45431, USA; 4 Department of Biomedical Engineering, University of California, Irvine, California 92697, USA; 5 Beckman Laser Institute, University of California, Irvine, California 92612, USA and Edwards Lifesciences Center for Advanced Cardiovascular Technology, University of California, Irvine, California 92697, USA; 6 Physics Department, Purdue University, West Lafayette, 47906, USA; 7 Department of Cellular & Integrative Physiology, Indiana University School of Medicine, Indianapolis, Indiana, 46202, USA

## Abstract

Lipid deposition inside the arterial wall is a key indicator of plaque vulnerability. An intravascular photoacoustic (IVPA) catheter is considered a promising device for quantifying the amount of lipid inside the arterial wall. Thus far, IVPA systems suffered from slow imaging speed (~50 s per frame) due to the lack of a suitable laser source for high-speed excitation of molecular overtone vibrations. Here, we report an improvement in IVPA imaging speed by two orders of magnitude, to 1.0 s per frame, enabled by a custom-built, 2-kHz master oscillator power amplifier (MOPA)-pumped, barium nitrite [Ba(NO_3_)_2_] Raman laser. This advancement narrows the gap in translating the IVPA technology to the clinical setting.

An unmet clinical need exists to detect unstable plaque in cardiovascular disease (CVD), the number one cause of death in the United States^1^. Vulnerable plaques have a high risk of rupture and thrombosis, which account for the majority of fatal acute coronary syndromes^2^,^3^,^4^,^5^. Currently, no imaging tools exist to reliably and accurately diagnose a vulnerable plaque in live patients^6^; instead, only autopsies can reveal ruptured lipid-laden thin fibrous cap atheromas^7^,^8^. Among the current interventional imaging procedures, intravascular ultrasound (IVUS) lacks the chemical selectivity to determine the composition of the vessel wall^9^, and the validity of IVUS image processing to achieve so-called “virtual histology” has been challenged^10^. Intravascular near infrared spectroscopy can detect lipids in the vessel wall^11^,^12^,^13^, but without depth-resolved spatial resolution. Intravascular fluorescence imaging is another emerging technique and it can identify the inflammation by visualizing exogenous contrast dye^14^, yet it suffers from the shallow imaging depth. Intravascular optical coherence tomography (OCT) accurately detects the surface layer of arterial wall with micron-scale resolution^15^,^16^, but has neither sufficient imaging depth nor chemical selectivity to determine plaque composition. Recently, a combined IVUS and OCT system was evaluated to demonstrate its co-registered dual-modality imaging capability of coronary arteries by providing the deep imaging depth of IVUS and high resolution of OCT^17^,^18^. Even though this combined technique carries the complementary morphological information from IVUS and OCT, it still has limited capability of assessing the plaque vulnerability due to the lack of chemical information. These challenges raise an unmet need for a novel intravascular imaging system which possesses chemical selectivity and depth resolution.

Photoacoustic (PA) endoscopy, which utilizes the optical absorption properties of tissue composition as contrast, could bridge the abovementioned gap. This technique, which applies pulsed light excitation, has been demonstrated for multiple endo-cavity imaging applications, such as esophageal and transrectal imaging in animal models *in vivo* using hemoglobin absorption as contrast mechanism^19^. Nevertheless, contrast based on electronic absorption, as in the case of hemoglobin, has limited tissue specificity within the arterial wall^20^. A new contrast mechanism based on the overtone absorption of C-H bonds excited by wavelengths of 1.2 or 1.7 µm provides lipid-specific PA contrast, and has been used for imaging lipid-laden plaque^21^,^22^,^23^,^24^. Efforts have been made to translate the concept of vibration-based PA imaging to a clinically relevant setting through the development of an intravascular photoacoustic (IVPA) system. A few groups have demonstrated the feasibility of imaging lipid-laden plaque^22^, even in the presence of blood^23^,^25^. However, the transition of such IVPA systems from bench to bedside has been stifled by its slow imaging speed. Current IVPA systems employ a commercial Nd:YAG-pumped optical parametric oscillator (OPO) system with 10 Hz repetition rate to generate the excitation at 1.7 µm and 1.2 µm wavelengths for lipid visualization^22^,^23^,^26^. This low repetition rate translates to a cross-sectional imaging speed of 50 s per frame of 500 A-lines, which is marginally useful for clinical applications.

Herein, we demonstrate a Ba(NO_3_)_2_-based Raman shifter (or Raman laser) pumped by a 1064 nm 2 kHz master oscillator power amplifier (MOPA) with a tunable pulse duration. Based on the principle of stimulated Raman scattering, the output wavelength of a Raman shifter is determined by the pump wavelength and the Raman modes of the medium of the shifter. In our study, we applied a Ba(NO_3_)_2_-based Raman shifter with a major Raman mode (Ω) at 1047 cm^−1^, to convert the 1064 nm pump to a 1197 nm output (Fig. 1 A). We employed a 2 kHz MOPA system as a pumping source and obtained an output of 2.0 mJ pulse energy at 1197 nm at 2 kHz repetition rate from the Raman shifter. The conversion efficiency of our Raman laser is 32%, which is ~ 1 order of magnitude higher that the commercially available OPO system. This high repetition rate laser system enabled the IVPA imaging of lipid-laden plaque with 1 Hz frame rate, which is nearly two orders of magnitude faster than the reported systems^22^,^23^,^26^.

## Results

### Characteristics of the Raman laser

**Figure 1:**
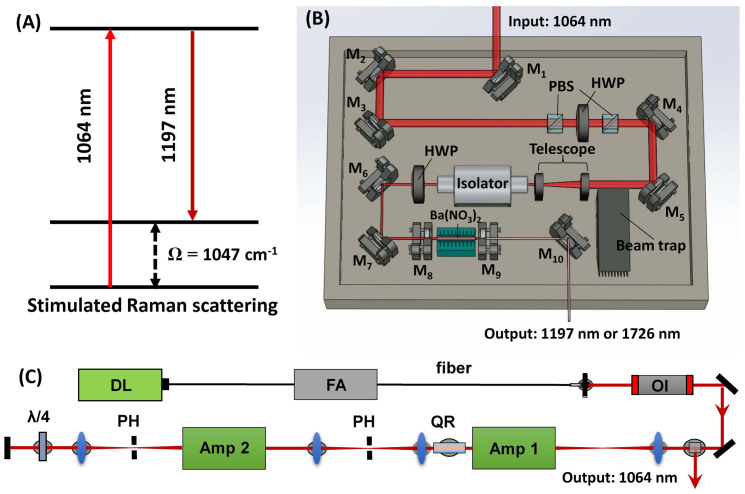
Principles and schematics of the Raman laser system. (A) The principle of the Ba(NO_3_)_2_ crystal-based Raman Shifter. (B) The Schematics of the Raman shifter: M_1_-M_7_: 45° 1064 nm reflective mirror; PBS: polarizing beam splitter; HWP: half wave plate; M_8_: resonator end mirror; M_9_: output coupler; M_10_: silver mirror. (C) The schematics of the MOPA system: Amp: amplifier; PH: pin hole; QR: quartz rotator; OI: optical isolator; FA: fiber amplifier; DL: Directly modulated diode laser.

We designed and constructed the Raman laser to generate 1197 nm excitation (Fig. 1 A) for the IVPA imaging by employing a Ba(NO_3_)_2_ crystal-based Raman shifter and a compact 2 kHz master oscillator power amplifier (MOPA) laser system with 2 kHz pulse train, high pulse energies, and the ability to control the pulse width (Fig. 1 B and C). Details of the lasers construction can be found in the Materials and Methods section.

**Figure 2:**
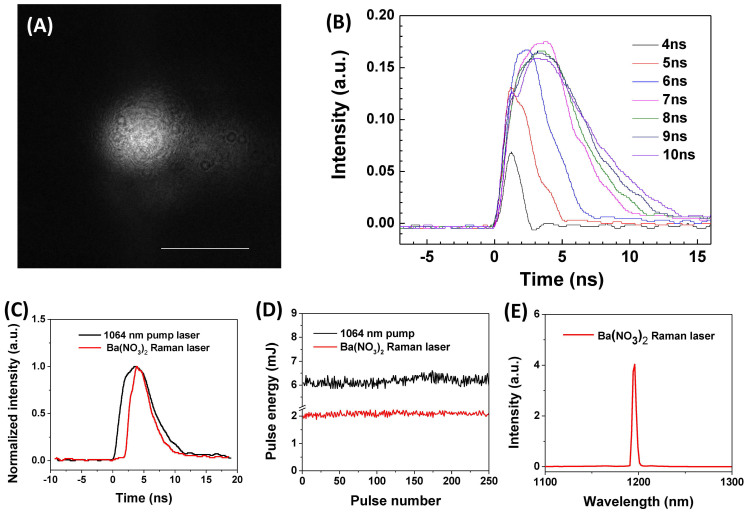
Specifications of the MOPA system and the Raman laser. (A) The beam profile of the MOPA system. Scale bar: 1 mm. (B) The pulse duration of the 1064 nm from the MOPA system with different trigger pulse duration. (C) Pulse duration of the 1064 nm pump laser (black) and the Ba(NO_3_)_2_ based Raman laser (red). (D) The pulse-to-pulse variation of 1064 nm pump laser (black) and the Ba(NO_3_)_2_-based Raman laser (red). (E) The output spectra of the Ba(NO_3_)_2_ based Raman laser.

We characterized the MOPA pumping source and the output from the Ba(NO_3_)_2_-based Raman shifter. The beam profile of the 1064 nm output from MOPA system is shown in Fig. 2 A. 90% of the energy is present in the Gaussian beam profile with a width (*1/e*^*2*^) of 1 mm. 10% of the total pulse energy was in the background originating from amplified spontaneous emission. Overall beam quality was measured to be M^2^ = 1.6. It is important to note that due to the non-linear nature of the stimulated Raman scattering process, the low power background was not Stokes shifted after passing through the Raman shifter, and thus it did not affect the output beam profile. This beam quality ensured that no hot spots were generated in the Raman cavity and thus reduced the risk of damaging the optics inside the Raman shifter. The pulse width of the 1064 nm output from MOPA system can be controlled by changing the pulse width (2 ns to 100 ns) of the directly modulated diode. For greater efficiency in generating photoacoustic signal, we controlled the pulse duration to be less than 10 ns. In Fig. 2 B, it is shown that by changing the duration of the trigger pulse generated in the pulse generator from 4 to 10 ns, the pulse duration of the 1064 nm output can be controlled in the range of 2 to 8 ns. From this result, we found that the peak intensity of the 1064 nm output started to drop when the trigger pulse duration is longer than 7 ns, which corresponds to the actual pulse duration of 5.8 ns. We then set the MOPA system to a pulse duration of 5.8 ns (Fig. 2 C, black line) to generate the output at the highest efficiency. The MOPA system generated 6.25 mJ pulse energy with a pulse-to-pulse variation of 2.6% (Fig. 2 D, black line). The output from the Ba(NO_3_)_2_-based Raman shifter has a pulse duration of 3.7 ns (Fig. 2 C, red line) and a 3.3% pulse-to-pulse variation (Fig. 2 D, red line) at 1197 nm (Fig. 2 E). At the maximum obtained output of 2 mJ at 1197 nm, the calculated conversion efficiency of the Raman shifter was 32%, which is slightly lower than the reported 34.8% efficiency for a high energy 10 Hz system^27^. The obtained pulse energy of a few millijoules at 1197 nm is comparable to what has been used for IVPA imaging^22^,^23^. The above data demonstrated that 1197 nm excitation with a nanosecond pulse duration can be achieved at a 2 kHz repetition rate, which is two orders of magnitude higher than that for the low repetition rate lasers used in current IVPA imaging systems.

The first Raman laser which implemented in photoacoustic imaging utilized Q-switched Nd:YAG pumping source^27^. The difference between the MOPA and Nd:YAG pumped Raman laser is summarized in Table 1. It is shown that MOPA pumped Raman laser has higher repetition rate, pulse-to-pulse stability, and beam quality, but lower maximum power. Those characteristics can be translated to high imaging speed, no need of pulse-to-pulse normalization and high fiber coupling efficiency. This result indicated that MOPA pumped Raman laser is suitable for microscopy and endoscopy applications, while the Q-switched Nd:YAG pumped Raman laser is suitable for tomography applications^28^.

### The IVPA imaging system

**Figure 3:**
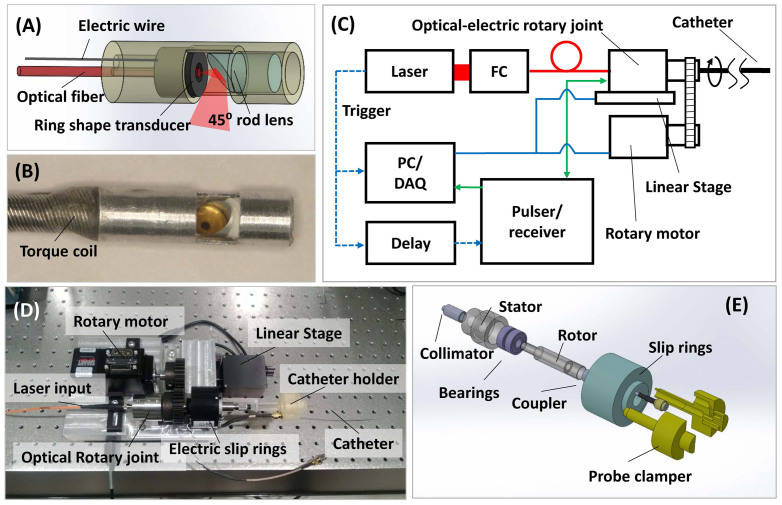
Schematics of the IVPA system: Schematic (A) and photograph (B) of the IVPA probe. (C) Block diagram showing the data acquisition system. FC: fiber coupler; PC/DAQ: personal computer, data acquisition. (D) Photograph of the scanning assembly. (E) Schematic of the assembly of optical rotary joint and electrical slip rings.

We have designed and developed an IVPA system (Fig. 3, details in the Materials and Methods section). In this system (Fig. 3 A and B), we applied a 35 MHz ring-shape transducer and a 400 μm core fiber, which was concentrically aligned with the hole of the transducer. Both the excitation light and ultrasound transmission were reflected by a 45 degree rod mirror at the tip of the probe. A torque coil was used to house the optical fiber and electric wire to provide the rotary torque directly to the tip of the probe (2.9 mm in diameter). To enable 2-dimensional (2-D) IVPA imaging, we developed a scanning interface, which included a customized optical rotary joint with electrical slip rings, a rotatory motor and a linear translation stage (Fig. 3 C and D). Our free-space optical rotary joint (Fig. 3 E) had a much higher damage threshold (> 10 W with 400 µm core fiber), as compared to the commercially available fiber-based optical rotary joint (< 1 W), in the near infrared region. Thus, it was suitable for transmitting the Raman laser with up to 10 W power. The insertion loss of the optical rotary joint was ~5 dB.

### High-speed IVPA imaging of lipid-mimicking phantom

**Figure 4:**
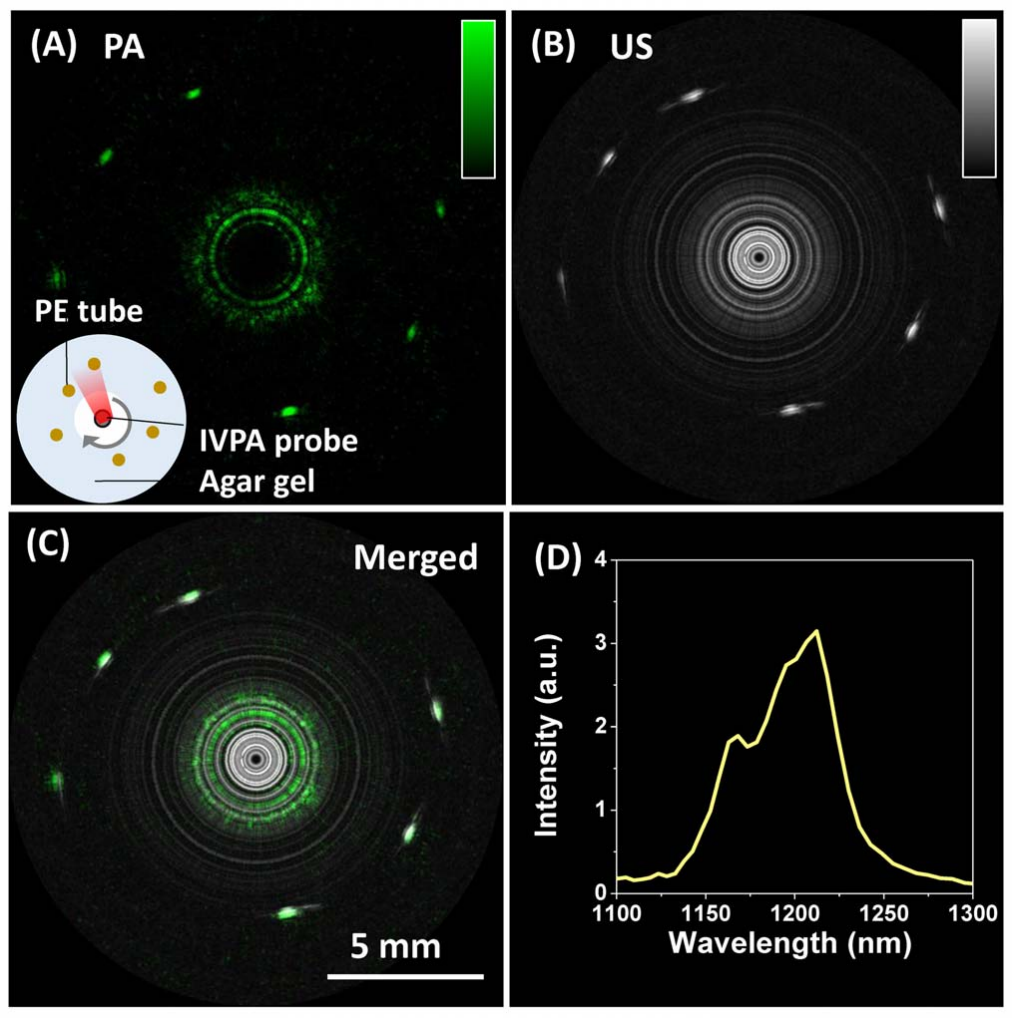
High-speed imaging of a lipid-mimicking phantom. Photoacoustic (PA) image (A), ultrasound (US) image (B) and merged image (C) of a polyethylene (PE) tube. (D) Photoacoustic spectrum of the PE tube.

We used 6 polyethylene (PE) tubes with diameter of 1.2 mm, which were scattered in a agarose gel, as a lipid-mimicking phantom to demonstrate the C-H bond-selective IVPA imaging using the Ba(NO_3_)_2_ –based Raman laser (Fig. 4 A–C). To perform co-registered photoacoustic (PA) and ultrasound (US) imaging, we delayed the US pulser 11 μs after the laser pulse to ensure that the PA signal and US signal would not overlap in time. The PA (Fig. 4 A) and US (Fig. 4 B) images were acquired sequentially, and the merged image (Fig. 4 C) confirmed the co-registration of PA and US images. 1000 A-lines were acquired for one 2-D cross-sectional image, resulting in a 2 Hz frame rate. Two adjacent A-lines were binned in each image. This speed is two orders of magnitude faster than previously reported IVPA systems^22^,^23^,^26^. PA spectroscopy of PE showed the signature peak of C-H overtone vibration band at 1210 nm, which confirmed that the contrast was indeed from C-H bond vibration (Fig. 4 D).

### High-speed PA imaging of lipid-laden artery

**Figure 5:**
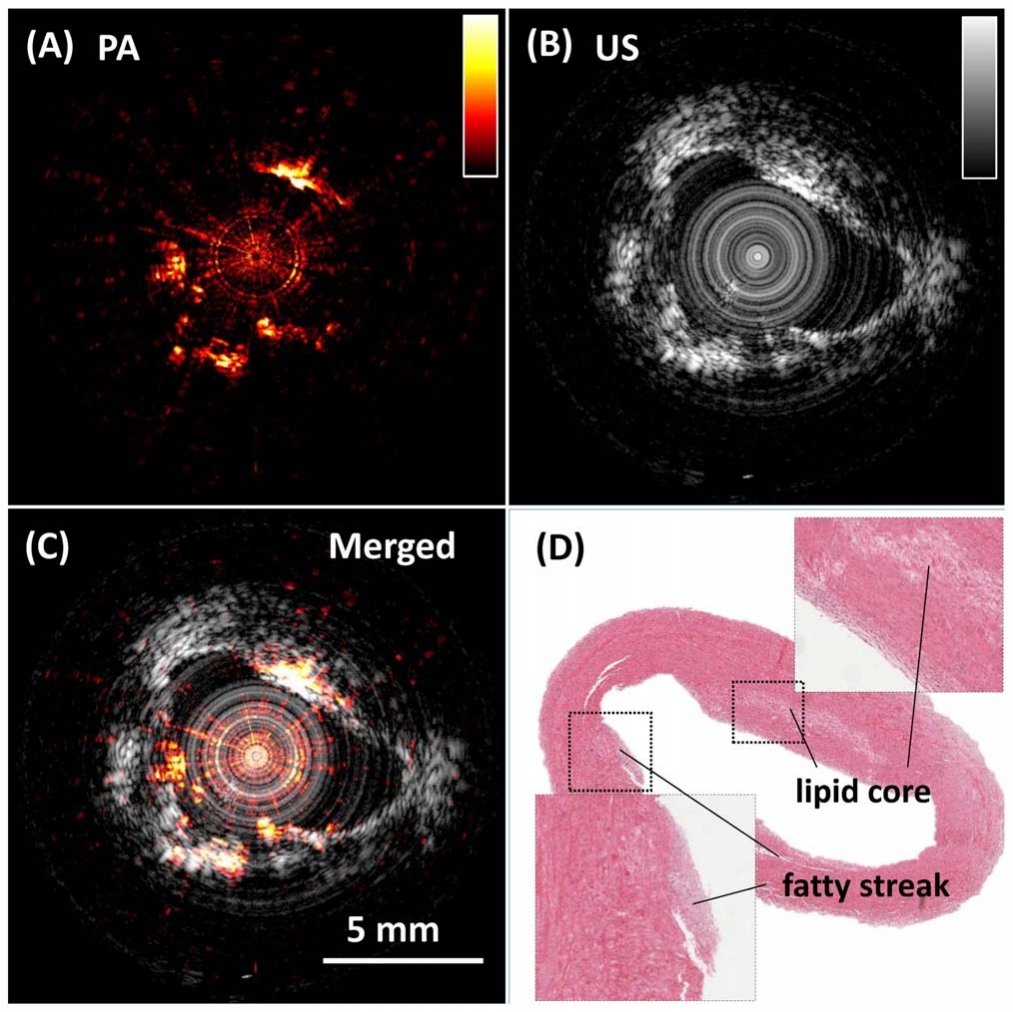
High-speed IVPA (A) and IVUS (B) imaging of an atherosclerotic artery. (C) Merged PA/US image. (D) Histology of the artery cross section of the area imaged by IVPA method. The 5 mm spatial calibration applies to all panels.

Using the 2-kHz Ba(NO_3_)_2_-based Raman laser, we further demonstrated high-speed IVPA imaging of an iliac artery from an Ossabaw pig with atherosclerosis (Fig. 5). Cross-sectional photoacoustic (Fig. 5 A), ultrasound (US) (Fig. 5 B), and merged (Fig. 5 C) images of the atherosclerotic artery clearly show the complementary information of the artery wall. Importantly, lipid deposition on the arterial wall, which is not seen in the US image, shows clear contrast in the IVPA image. The white area in the histology image from hematoxylin and eosin staining shows the location of the lipid deposition (Fig. 5 D). The white color is caused by the washout of lipid during the paraffin embedding process. This *ex vivo* artery was not pressure-perfused during sacrifice, and thus the lumen is partially collapsed. 2000 A-lines were acquired for the cross-sectional image, resulting in a 1.0 Hz frame rate. 4 adjacent A-lines were binned in each image. The pulse energy was 80 µJ. The energy density at the surface of the probe was calculated to be 4 mJ/cm^2^, which is below the ANSI laser safety standard (20 mJ/cm^2^ for nanosecond laser at 1200 nm)^29^.

## Discussion

In this work, we constructed a Ba(NO_3_)_2_-based Raman laser generating the wavelength at 1197 nm and demonstrated its use for PA mapping of lipids deposited within the artery wall. It is known that both excitation wavelengths at 1.2 and 1.7 µm are resonant with overtone vibrations of the C-H bond, which can provide the lipid-specific contrast^25^. Studies have shown that 1.7 µm is the best wavelength for intravascular imaging due to the high absorption coefficient and less scattering by blood^23^,^25^. However, considering the optical absorption by water, 1.7 µm is not the optimal excitation wavelength in all cases. It has been demonstrated that if the excitation light travels more than 4 mm to reach the lipid deposition inside the artery, it is better to use 1.2 µm excitation due to less excitation attenuation by water absorption^25^. In our experimental configuration, the inner diameter of the probe is ~3 mm, but the distance between the fiber tip and the mirror reflector is ~ 1.5 mm. Thus there is a ~3 mm space between the fiber tip and the surface of the probe, over which the excitation light traveled. Moreover, considering the gain in depth information through the arterial wall (>2 mm), we chose to use 1.2 µm excitation to avoid attenuation from water and to obtain the best signal inside the arterial wall. However, for a smaller size transducer and coronary artery applications, it is still better to use 1.7 µm excitation for the imaging of lipid-laden plaque.

In this study, we employed a single-color Raman laser for IVPA imaging of lipids. Compared to fibrous or smooth muscle tissue, a lipid core has an over ten-fold increase in C-H bond density. This contrast has proved to be high enough to discriminate a lipid core from other tissue. Further, the thin, mildly echogenic slice in the absence of peripheral acoustic shadowing at about 1 o'clock in the image is classified as “fibrous” in typical IVUS descriptions^30^.

It is reported that calcified tissue produces a PA signal as well. This type of tissue represents the most significant contrast ambiguity we are facing^31^. However, since IVPA imaging will be performed at the same time as IVUS imaging, the IVUS image will help to clarify this ambiguity. IVUS provides a very distinct echogenic signal with peripheral acoustic shadowing from calcification^30^. Meanwhile, we note that multi-wavelength imaging has been proved to be an effective way to differentiate tissue types in arterial tissue^31^,^32^. Applying an Ytterbium-doped laser with 1073 nm wavelength as the pumping source, it is possible to build a Raman laser outputting wavelength at 1210 nm^33^. With 1197 nm and 1210 nm excitation, which corresponds to the peak for second overtone of CH_2_ and CH_3_, respectively^25^. This would enable imaging of lipid and protein in the arterial wall to further characterize the fibrous cap (largely collagen and elastin) and the precise thickness of the lipid core. This is highly significant for diagnosis of vulnerable plaque, since it has been generally accepted that a large lipid core representing >40% of the total atherosclerotic lesion area is a prominent characteristic^8^.

We note that intravascular OCT is well developed^15^,^16^, having 10–20 µm resolution, which could enable quantitation of the fibrous cap thickness. Since a critical parameter of plaque vulnerability is a thin fibrous cap of 65–80 µm^8^, OCT measures are superior to IVUS and IVPA in that regard. However, the spatial resolution of OCT is offset by the poorer depth of penetration through blood and tissue^6^. Further, continuous flushing of blood from the arterial lumen is required even with frequency domain OCT, which can adversely impact the electrocardiogram during imaging. IVPA technology is comparable to OCT in the compatibility with IVUS^16^, especially because IVPA and IVUS share the same detector and can be performed simultaneously using the same catheter. The most important advantage of IVPA is that it provides a direct, chemical measure of the other major component of vulnerable plaque – lipid-rich pools in the vascular wall^8^. In contrast, OCT imaging of lipids is considered largely subjective with poor accuracy in defining boundary^34^. Integration of OCT with NIRS and NIRF has been proposed to overcome this limitation^15^. However, NIRS and NIRF do not have depth sensitivity, and cannot provide tomographic image. Imaging intravascular lipid in vivo with IVPA, simultaneously with IVUS, may allow physicians to better evaluate the vulnerability of the atherosclerotic lesion.

The MOPA system with its high beam quality played an essential role in our development. Owing to the utilization of a polarization-maintaining single-mode fiber in fiber amplifier before the free space amplifiers, the beam quality of 1064 nm output from the MOPA was measured to be M^2^ = 1.6. This high-beam quality ensured the high conversion efficiency of the Raman shifter. Moreover, the pulse duration of the MOPA system is tunable, and it is known that the bandwidth of the photoacoustic response is dictated by the optical excitation pulse duration and the relaxation response of the sample. Although the general paradigm for photoacoustic imaging is that the optimal pulse duration is around 5 to 10 ns, the optimal pulse duration for IVPA imaging using a high-frequency transducer depends on many factors, including the penetration depth. This pulse-duration-tunable MOPA system can potentially provide optimization of the signal for different sample conditions.

In summary, by design and development of a kHz repetition rate Raman laser, we have improved the intravascular photoacoustic imaging speed by two orders of magnitude. The reported advancement represents an important step to facilitate translation of intravascular photoacoustic technology to clinical applications.

## Methods

### Raman shifter

Schematic of the Raman shifter cavity is shown in Fig. 1 B. The Ba(NO_3_)_2_ crystal (Del Mar Photonics, California, USA) was installed in a flat-flat resonator with a cavity length of about 10 cm. For optimal first Stokes generation, the resonator's end mirror was coated with anti-reflectivity (AR) at 1064 nm, and high reflectivity (HR) at 1197 nm. The output coupler was coated with high reflectivity at 1064 nm, and 40% transmission at 1197 nm. The Ba(NO_3_)_2_ crystal was AR coated at 1064 nm and 1197 nm.

### MOPA system

MOPA pump laser with tunable pulse duration (Spectral Energy LLC, Ohio, USA) was employed as the pumping source, which provides up to 7 mJ pulse energy with a 2 kHz repetition rate with an arbitrary pulse duration. The optical layout is shown in Fig 1 C. The output of a directly modulated diode laser (1064.4 nm vacuum wavelength, 100 mW peak power) is first preamplified in a 30 dB ytterbium fiber amplifier before being amplified in two diode pumped Nd:YAG amplifiers arranged in a double pass configuration. Lenses L_1_ and L_4_ with a 50 mm focal length were used to compensate for thermal lensing in the rods. To minimize the depolarization effect the quartz rotator was placed between two amplifier modules. In addition, to minimize the amplified spontaneous emission the pinholes were placed at a focal plane of optical relay composed of lenses L_2_ and L_3_ and at the focal plane of second amplifier module. After double passing amplifier modules the beam is directed into the Raman shifter.

### IVPA catheter fabrication

A 35 MHz ring-shape transducer with 65% bandwidth and 3 mm focal length was applied by using mechanically press-focusing technique. The new generation single crystal Pb(Mg_1/3_Nb_2/3_)O_3_-PbTiO_3_ (PMN-PT) was chosen as functional piezoelectric material based on its superior electromechanical coupling coefficient^35^. The ring-shape transducer had a 0.5 mm center hole with which a 400 μm core multimode fiber (Thorlabs, NJ) was concentrically aligned. This design ensured that the US pulse and optical excitation were aligned during the procedure. Both the excitation light and the ultrasound transmission were deflected by a 45 degree rod mirror (Edmund Optics, NJ) with 2 mm size at the tip of the probe. A torque coil was used to house the fiber and electric wire and transmitted the torque directly to the tip of the probe. The size of the probe is 2.9 mm in diameter.

### Scanning system, data acquisition system and data processing

We assembled a mechanical scanning system by integrating an optical rotary joint with electrical slip rings (Moog, Inc, CA) (Fig. 3 C). The optical rotary joint was designed and constructed under the concept of free space coupling with a large beam size to avoid damage to the optics. The distal end of the fiber and electrical wires were connected to a fiber optical rotation joint and electric slip-rings, respectively. The catheter was driven by a computer controlled servo motor (Moog, Inc. CA) at the desired number of rotations per minute, while the outer housing remained stationary. The video in the Supplementary Information shows that the scanning system is rotating at 60 rotations per min while still transmitting the optical excitation. The trigger of the laser was used to synchronize the data acquisition of both PA and US imaging. A delay generator was used to set an 11 μs delay between the laser pulse and pulser/receiver (5073PR Olympus, Inc). The detected PA signal was preamplified and then sent to a receiver. The total amplification was set at 69 dB. The signal was digitized and recorded by a PC. A digitizer with 180 MS/s sampling rate and 16-bit resolution (AlazerTech, Canada) was applied to enable high speed data acquisition and transfer. A modern i7 quad-core processor was employed for data processing. The data acquisition software was developed in LabVIEW. Data analysis was performed off-line by Matlab. A 20 MHz digital high-pass filter was applied for US images, while a 10 MHz digital high-pass filter was applied for PA images. The PA spectroscopy on PE tubes was performed by a Nd:YAG pumped OPO system (Surelite, Continuum, CA).

### Arterial tissue

The iliac artery specimens were harvested from an Ossabaw miniature pig that had diet-induced atherosclerosis that we have extensively characterized^36^. Iliac arteries were harvested and then preserved in 10% phosphate-buffered formalin. Before imaging was performed, arteries were washed in PBS for luminal imaging.

## Author Contributions

P.W., T.M., M.N.S. and S.L. contributed equally to the work. P.W., T.M., M.N.S. and S.L. performed the experiment and desgined the system. M.M.S. and S.R. contributed in the construction of the laser. M.S. provided the artery sample and provided advice on physiology. J.X.C., Z.C. and Q.Z. contributed on design of the experiment. T.M., Q.Z. and K.K.S. provided the transducer. S.L. and J.H. made the optical probe. All author contributed to the manuscript writing and review.

## Supplementary Material

Supplementary Information IVPA device

## Figures and Tables

**Table 1:** Comparison between the Q-switched Nd:YAG pumped Raman laser and the MOPA pumped Raman laser

	MOPA pumped	Q-switched Nd:YAG pumped
**Repetition rate**	2000 Hz	10 Hz
**Pulse-to-pulse variation**	3.3%	12%
**Beam quality (m^2^ factor)**	1.6	11
**Conversion efficiency**	32%	34.8%
**Maximum pulse energy**	2 mJ	105 mJ
**Pulse duration**	2–100 ns (tunable)	5 ns (fixed)
